# The experience of Family and Community Midwives in postnatal care: A qualitative study in Italy

**DOI:** 10.18332/ejm/204272

**Published:** 2025-05-26

**Authors:** Cristina Lumia, Irene Molinari, Beatrice Mazzoleni, Antonella Nespoli

**Affiliations:** 1 School of Medicine and Surgery, University of Milano-Bicocca, Monza, Italy; 2 Department of Biomedical Science, Humanitas University in Milan, Milan, Italy

**Keywords:** qualitative research, midwifery, postnatal, mother–baby dyad, family, continuity of care

## Abstract

**INTRODUCTION:**

Historically, national and international policies have focused more on care during pregnancy and childbirth rather than on the postnatal period. To solve the necessity of ensuring continuity of care after childbirth, the role of Family and Community Midwives (FaCM) was established permanently. The aim of the study is to explore the experiences and perspectives of the FaCM working in the Lombardy region of Northern Italy.

**METHODS:**

The method used in this qualitative study design is a descriptive phenomenological approach. The data collection was conducted through semi-structured interviews with FaCM who voluntarily indicated their willingness to participate in the study from January to March 2024. The interviews followed a topic guide based on available literature.

**RESULTS:**

Fifteen FaCM participated in the interviews. Two main themes emerged: 1) The contribution of a committed midwife in the community setting; and 2) The relationship between midwives, women, and other professionals. The contribution of a committed midwife in the community setting emphasized the novel role played by FaCM and their experience staying in connection with women after childbirth. Furthermore, midwives are the designated professionals responsible for ensuring continuity of care during the postnatal period. Relationships between midwives, women, and other professionals involved the challenge of collaborating within a team instead of working alone in a local service.

**CONCLUSIONS:**

The findings reflect the experience of FaCM in an entirely new postnatal community care setting. Enhancing the contribution of midwives alongside mothers and other professionals positively impacts the health of mothers and infants throughout the whole postnatal experience.

## INTRODUCTION

The postnatal period begins immediately after the birth of the baby and extends up to six weeks (42 days) after birth^1^. The main goals of postpartum and postnatal care are to protect and improve the health of the mother and infant while developing an environment that helps and supports the extended family and community to meet various health and social requirements^1^. Home-based postnatal care is suitable for healthy families, and it is essential to distinguish between healthy families and those requiring hospital care. This may signify an alternative to standardized durations in the maternity ward^2^.

A positive postnatal experience is characterized by the consistent provision of information, reassurance, and support from dedicated health professionals to women, newborns, partners, parents, caregivers, and all families; it occurs within a well-resourced and adaptable healthcare system that acknowledges the needs of women and infants while respecting their cultural contexts^1^.

Positive postnatal care is crucial to prevent adverse maternal and neonatal outcomes and to provide support during adjustment to motherhood, especially for new mothers^3^.

Women’s health throughout their entire lives has consistently been a priority for intervention, reflecting the quality of social and health services, as well as the significant value derived from the successful implementation of prevention and health promotion programs^4^. Health promotion campaigns, endorsed by World Health Organization (WHO)^5^, are based on active provision and empowerment and are characterized by their impact, which extends to the whole family and community. The WHO reports^1,5^ highlight that the key strategy for achieving the sustainable development goals for reproductive, maternal, and child health is to invest and focus on postnatal care.

Interventions and initiatives planned to enhance postnatal care services need to consider the involvement of competent midwifery staff. Care provided by the midwife during the early postnatal period is essential in building the woman’s trust in her ability to assume her new responsibilities as a mother. Women typically desire to converse about their childbirth experience, ideally with the midwife who attended the birth. Promoting relational continuity models of midwifery care is crucial to addressing the emotional dimensions of the postnatal period^6^. In Italy, midwives already provide postnatal care. However, community services may be different between national regions^7^. The national introduction of Family and Community Midwives (FaCM) in 2023 was set to significantly transform postnatal care, with a bill designed to enhance the quality of care for new mothers in Italy. This model is influenced by the directives of the National Institute for Health and Care Excellence (NICE), aiming to deliver continuous home care, minimize superfluous treatments, and enhance the connection between hospital and community^8^. The Lombardy area, located in Northern Italy, was among the first regions to implement a pilot project for the adoption of FaCM.

While there is extensive literature investigating women’s perspectives on the experience of postnatal care, there is a lack of literature exploring the views and experiences of Family and Community Midwives who provide this service^9,10^. The Midwifery Care Model^11^ highlights the importance of the FaCM, ensuring continuity of care within the communities in which women reside. In fact, it provides a first-level care system located close to women, de-medicalized but professional, offered by midwives to all women, newborns, and families^11^.

The aim of the study was to explore the experiences and perspectives of Family and Community Midwives (FaCM) working in the Lombardy region of Northern Italy. The experiences of the FaCM provided a description of their current extensive care actions in the community beyond the hospital.

## METHODS

### Study design and setting

A qualitative descriptive phenomenological^12^ study was conducted through semi-structured and face-to-face interviews with the study participants. Phenomenology is a philosophical theory, methodology, and approach applied to qualitative research that describes participant experiences, emphasizing the individual’s lifeworld^13^. Phenomenology presents challenges for researchers as it requires the perspectives of others’ perceptions, requiring the researcher to fully articulate this given without modification^14^. The interviews were conducted online (on Google MEET^®^ or Microsoft Teams^®^ platforms chosen by the participant). The experiences of Family and Community Midwives in the Lombardy region were described with the help of a specific topic guide based on the available literature.

### Study participants and ethics

Pilot study interviews were carried out through snowball sampling: three FaCM were enrolled. Data were omitted from the final analysis as the pilot interviews aimed to familiarize the researcher with the method. Participants in the FaCM pilot interviews were informed of the purpose of their participation and the opportunity to suggest improvements to the topic guide. Nevertheless, the topic guide remained unchanged without any modifications.

The selection of the main study participants was conducted using the convenience sampling strategy. The study group was recruited via an invitation letter distributed to Family and Community Midwives through the Orders of Midwives in the Lombardy region.

FaCM were included in the study from 22 January and 5 March 2024. The invitation letter enclosed the personal data of the researchers involved (email and telephone contact) through which they were contacted by the midwifery staff who voluntarily decided to participate in the interviews. Subsequently, upon agreement with the FaCM, based on their availability, the date and time of the interview were decided. Then, the connection link for the interview, the Information Form, the Informed Consent, and the Form for Consent to the Processing of Personal Data for Scientific Purpose were communicated.

First, to contextualize the results of the study, participants were surveyed regarding their years of experience as FaCM, the city and, the context in which they work, age, and gender.

There were seventeen midwives who expressed their consent to the interview. Two candidates withdrew their participation in the study before carrying out the interview for declared family reasons.

### Inclusion and exclusion criteria

FaCMs were included in the study if they met the following inclusion criteria: 1) Italian native language or well understood and spoken; and 2) At least 2 years of work experience as FaCM. No exclusion criteria were selected.

### Data collection

Interviews were audio recorded and transcribed verbatim.

To guarantee the protection of personal data, anonymization was applied to the data by assigning a participant code (MID01, MID02, etc.) to each transcription and the removal of personal names and places that were involuntarily mentioned during the interview. The collection of midwives’ availability was interrupted when, after interviewing 15 midwives, data saturation was reached.

### Data analysis

Data were organized and processed utilizing NVivo14^®^ software and analyzed employing the content analysis technique within the thematic analysis framework^15^. This technique involves a preliminary study of the data through multiple readings, the establishment of the corpus, and the formation and revision of hypotheses and objectives, followed by the examination, processing, and interpretation of the results collected.

## RESULTS

From the analysis of the data, it emerged that the sample had an average work experience within FaCM of 4.7 years (range: 2–10 years) and a mean age of 37 years (range: 28–51 years) (Table 1). The participants were all female. Interviews had an average duration of 32 min (range: 19–52 min)

**Table 1:** Participants’ information, a qualitative study on the experience of Family and Community Midwives (FaCM) in postnatal care, Italy, 2024 (N=15)

*Participant code*	*Date of interview*	*Duration of interview (min)*	*Age (years)*	*Work context*	*Years of experience within FaCM*	*City*
MID01	22/01	39	32	Accredited services linked to the Public Assistance	8	Cremona
MID02	23/01	19	51	Italian National Health Service	2	Varese
MID03	24/01	31	39	Italian National Health Service	2	Milano
MID04	29/01	29	43	Italian National Health Service	4	Milano
MID05	30/01	27	28	Italian National Health Service	2	Varese
MID06	06/02	29	50	Italian National Health Service	6	Bergamo
MID07	08/02	32	39	Italian National Health Service	4	Bergamo
MID08	12/02	52	28	Italian National Health Service	5	Como
MID09	14/02	45	28	Accredited services linked to the Public Assistance	4	Como
MID10	26/02	34	33	Accredited services linked to the Public Assistance	10	Como
MID11	26/02	31	44	Freelance	5	Varese
MID12	27/02	28	33	Accredited services linked to the Public Assistance	7	Como/Lecco
MID13	29/02	21	34	Accredited services linked to the Public Assistance	2	Como
MID14	04/03	33	32	Freelance	7	Monza-Brianza
MID15	05/03	27	43	Italian National Health Service	3	Milano

Two themes and four sub-themes were identified. Firstly, the contribution of a committed midwife in the community setting was emphasized by FaCM. The adoption of a community model of midwifery care brings midwives closer to women and infants. Midwives revealed enhanced expertise in health and birthing knowledge. The second theme accentuates the interaction between FaCM more than ever in this community context. Consequently, collaborating within a multidisciplinary team was challenging in an innovative care environment. The FaCM were always available also for unexpected needs.

### The contribution of a committed midwife in the community setting

The first thematic area aims to explore the impact and experience of the FaCM service following its introduction within the community setting. Family and Community Midwives are essential to ensuring continuity of care post-hospital discharge. This novel service begins with an appointment of staff within FaCM to oversee the mother–baby dyad soon following birth. The hospital, after obtaining the women’s informed consent, transmits the names of recent mothers to the community midwife. FaCM have a role and a responsibility to contact these women for a postpartum visit at least 24 hours after birth. In certain instances, a sole midwife is designated to do telephone triage to assess the mother and guide her to the right local service provider. In these situations, the midwife’s responsibility is to promptly understand a need, ensuring the woman does not feel abandoned following hospitalization. A midwife’s role is to promptly identify specific vulnerabilities and to proactively assist the mother:

*‘If hospital colleagues send a report of a vulnerable nucleus, we contact the woman and offer a home visit.’* (MID15)

*‘The certainty that someone will arrive at your home as soon as you arrive from the hospital, when there is a moment of panic, is truly very useful.’* (MID11)

At the end, midwives interviewed supported the importance of continuity of care, both to foster a relationship of trust with the woman and because this would allow us to identify any problems, which would not emerge with a single visit.

### Midwives at the right place and at the right time

FaCM emphasizes the timing for a caring contact with the mother–baby dyad, a guaranteed communication that was previously unreachable without midwives in community services. Interviewed midwives affirmed that a visit by a midwife within FaCM is planned for all mothers within 48–72 hours post-discharge or within 14 days following childbirth. In instances of obstetric or neonatal history, a color code is designated to the dyad by the hospital midwives via the completion of a triage form. The color code is designated based on the severity of the assistance required. Red indicates a critical situation necessitating immediate assistance, whilst green signifies a deferrable condition:

*‘If the code is red, a mother who is discharged with artificial breastfeeding because the baby is premature, they receive a visit the next day, maximum 24/48 hours’*. (MID03)

*‘I remember of a woman with a history of preeclampsia who told us that the midwives are always in the right place in the right time. She was worried about her blood pressure at home.’* (MID01)

Regarding the presence of barriers that limit postnatal care at home: FaCM stated that the main limitation is the lack of midwife staff. Midwives are necessary to guarantee the home visiting service to all mothers who have recently given birth. It was also revealed by MID12 that the most important barrier is the lack of knowledge of the community service because hospitals offer postnatal care services instead of referring the dyad to FaCM.

Midwives facilitate women’s empowerment throughout the birthing process, and the personal decision about using community services was another theme that surfaced during the interviews:

*‘Asking the women to call us makes them more responsible for what they need. And yes, I like this because we empower at all stages of women’s experience of childbirth.’* (MID5)

### The midwife’s task in sharing information regarding the mother and the newborn

Another sub-theme that developed from the interviews was to recognize the importance of information exchange between the inpatient facility, where the birth has taken place, and the community service. The FaCM are in charge of investigating the mother–baby history documented in the discharge letter. In addition, midwives of the hospitalization facility also report the most relevant clinical and social information on the triage form, already cited before. They perform this task because they are aware that a colleague will undoubtedly review the hospital report. Woman’s story of her experience is not sufficient to reconstruct the clinical history:

*‘In the form there is a small final part with name and surname, address, telephone number. Below there are notes and if there is a special case, they write there.’* (MID15)

The interviewed midwives underlined the necessity of augmenting home visits and asserted that all women should be informed about the newest postnatal care services available in the area during their pregnancy, ensuring they know whom to contact after discharge. MID03 emphasized the necessity of identifying the instruments and resources required to ensure home visiting services for non-EU women through coordination with the currently inactive cultural mediation service.

### Relationship between midwives, women and other professionals

The second thematic area concerns the relationship between new mother and FaCM.

The interviews provided a summary of the services offered through community services and the role of midwives in promoting a nurturing relationship for women and newborns (Table 2).

**Table 2:** Services offered in postnatal care from the interviews, a qualitative study on the experience of Family and Community Midwives (FaCM) in postnatal care, Italy, 2024 (N=15)

*Services*	*Participants*														
	*1*	*2*	*3*	*4*	*5*	*6*	*7*	*8*	*9*	*10*	*11*	*12*	*13*	*14*	*15*
Home visiting	x	x	x	x		x	x	x	x	x	x	x	x	x	x
Local visiting	x	x	x	x	x	x	x	x	x	x		x	x	x	x
Individual counseling for breastfeeding	x	x	x	x	x	x	x	x	x	x	x	x	x	x	x
Telephone counseling/telemedicine	x	x	x	x	x	x	x	x	x	x	x	x	x	x	x
Puerperium meeting	x				x			x	x	x					
Free access meeting		x	x		x	x		x	x	x		x	x		x
Perineal rehabilitation	x							x	x			x			
Weaning	x		x		x	x	x	x	x	x		x			x
‘Born to read’ initiative				x	x	x	x		x				x	x	
‘Born for the music’ initiative/music therapy				x					x			x			
Infant massage course	x			x		x	x	x	x	x		x	x	x	x
Baby wearing				x					x						
Meeting dedicated to fathers	x					x	x	x							
Meeting dedicated to grandparents								x							
Pediatric airway unobstruction course	x		x			x	x								

FaCM agreed in maintaining the issues that need to be more integrated with the dyad at first contact, like breastfeeding and lactation physiology and the care of the newborn:

*‘The fear of the child not growing or about breastfeeding, there’s still a lot of work to be done on that!’* (MID07)

*‘Recognizing the difficulties of postnatal care for women lacking guidance at critical moments is not simple. Childbirth extends beyond the confines of the delivery room.’* (MID08)

### Working in a team is challenging

The FaCM interviewed shared their experience of working in a team outside the hospital setting for the first time. In the Italian context, it has been challenging to establish a team consistently available outside of extensive care centers:

*‘Previously, working in a “Consultorio” (community and local service in Italy) sometimes entailed isolation, with a doctor present only sporadically each week, while mental health services were infrequently accessible and challenging to obtain.’* (MID03)

Certain people, including social workers, were conspicuously absent:

*‘Now, the social assistant is dedicated and most closely to the local and community network. I feel more comfortable knowing that if there is a specific call for a consult, someone may provide immediate assistance to our mothers.’* (MID09)

As regards the presence and collaboration of other professional figures, FaCM who practice in community services have reported that a psychologist, a social worker, a gynecologist, a nurse, or a health assistant, are present in order to guarantee an integrated multidisciplinary approach. Continuity of care provided by other professionals makes the midwifery care more efficient and safe. In some areas there is also a pedagogue and administrative staff.

**Figure 1:**
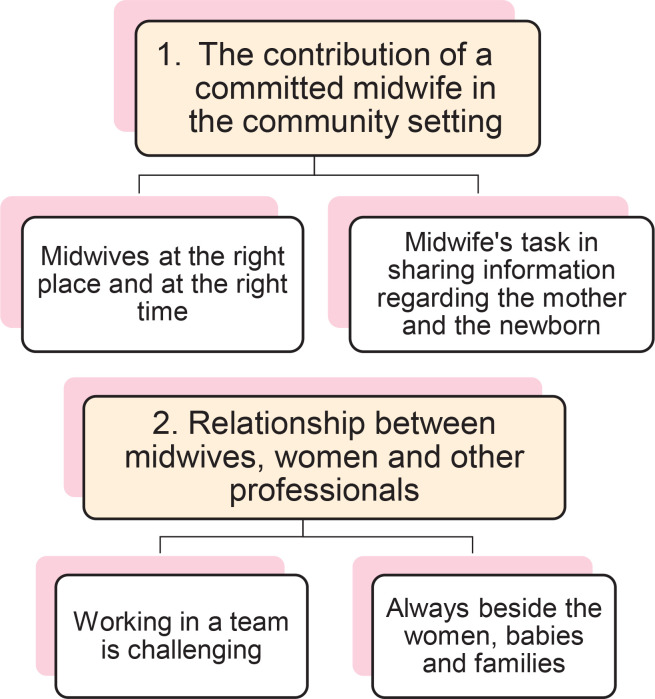
Themes and sub-themes from the interviews, a qualitative study on the experience of Family and Community Midwives (FaCM) in postnatal care, Italy, 2024 (N=15)

### Always beside the women, babies and families

The final sub-theme highlighted the importance of staying present for all families. Community services are available Monday to Friday, from 8:00 a.m. to 4:30 p.m., with postnatal care services accessible outside these hours on an on-call basis in urgent situations:

*‘It happened even on Easter and Christmas if red codes arrive.’* (MID01)

*‘The colleagues at the hospital are consistently engaged; I also found it gratifying to respond to a call on a Sunday morning. My personal time is irrelevant, the needs of women in crisis take precedence. Is this not the core of our activity?’* (MID13)

## DISCUSSION

The contribution of a committed midwife in the community setting and the relationship between midwives, women and other professionals are the principal findings of this study. The discussion is categorized into two subheadings, guided by the existing literature: 1) The role of the FaCM for the continuity of care; and 2) Midwives working in a team in the community settings.

### The role of the FaCM in the continuity of care

In numerous resource-limited contexts, trained community health workers frequently serve as the initial interface between communities and the healthcare system^16^. They are crucial in transmitting key health information in a culturally sensitive way, enabling community members to make informed decisions along with improving community-level access to vital health services^17^.

The largest number of midwives predominantly work inside hospital settings, with few involved in community-based practice^18^. In Italy, to promote the establishment of postnatal services, a professional post-degree program has been established to generate new employment opportunities and deliver evidence-based practices: Family and Community Midwifery^19^.

It is recognized that women under the care of midwifery group practices get improved prenatal and postnatal care, accompanied by notable cost savings^20^. The need for the implementation of hospital and community midwifery care continuity models was reiterated by the midwives interviewed. As stated by Crowther et al.^21^, planned midwifery care, combined with models of continuity, represents an opportunity to improve the postnatal experiences of women and families, because they can offer to the dyad a dynamic and personalized care experience.

The continuity of care after hospital discharge is essential. The Italian context examined by the FaCM participants somewhat aligns with WHO recommendations^1,5^: mother and baby should get a home visit within 24 hours. Participants of the study schedule an evaluation in 24 hours only for high-risk women or if there exists a specific requirement. However, one visit is planned for all mothers within 48–72 hours post-hospital discharge.

The role of FaCM is innovative and frequently inadequately incentivized. A French population-based retrospective study^22^ illustrates the importance of enhancing information quality and accessibility for professionals, particularly midwives, as effective strategies to boost the utilization of community postnatal care among women.

The experiences of the Italian FaCM interviewed demonstrate that when midwives participate in shared decision-making with mothers and provide consistent information, satisfaction with care increases^23^. An assessed postnatal care model in Sweden concluded that both midwives and new mothers reported positive experiences, considering the model attentive to their expectations^24^.

A previous qualitative study^6^, with the aim of investigating the fundamental importance of midwifery care during home visits in the postnatal period, has highlighted how women want to be recognized and given time to discuss their own experience of childbirth, also demonstrating a feeling of vulnerability in the new maternal role, especially regarding breastfeeding; it concludes by identifying the midwife as the professional responsible for welcoming these maternal emotions. As stated by Davis and Walker^25^, midwives work to strengthen a woman’s confidence in her ability to birth, grow and care for her baby. The midwife–woman relationship is the vehicle through which trust is built, personalized assistance is given, and the woman feels valued. Even so, it is strongly recommended not to always work alone.

### Midwives working in a team in the community settings

The improvement of postnatal care required a multidisciplinary approach due to the complexity of the demands of new mothers. A multidisciplinary team promotes the quality and efficacy of care by collaboratively integrating various healthcare specialists, resulting in more comprehensive assistance. The experiences of midwives underscore the significance, effectiveness, and methodologies for establishing multidisciplinary teams in community care.

As reported in a review^26^, midwives work collaboratively as part of a multidisciplinary team, providing integrated care in a community setting and effective care to women and newborns who develop complications. However, as shown in a qualitative study in the Netherlands, working in small teams in community midwifery enhances the quality of continuing care^27^.

Competent teamwork and collaboration may improve patient outcomes and job satisfaction for midwives^28^. In community settings, interactions are especially important, as midwives frequently serve as primary caregivers, engaging with obstetricians, nurses, and other professionals^29^.

The World Health Organization (WHO) and the National Institute for Health and Care Excellence (NICE) recommend the use of multidisciplinary teams in postnatal care, asserting that these strategies improve care quality and fulfill both physical and psychological support requirements^30^. Additionally, an interdisciplinary approach may mitigate the risk of overlooked or postponed diagnoses of postpartum disorders^31^.

According to the FaCM interviewees, having a physical presence at vulnerable moments is important. Indeed, preliminary screening and assistance have demonstrated efficacy in alleviating risks linked to emotional distress and developmental issues in the initial months of a child’s life^32^.

An extra issue that requires discussion is non-EU women, as suggested by FaCM. The seamless integration of care from several disciplines facilitates a more personalized approach to postnatal difficulties, as evidenced by studies on culturally sensitive care for non-Western immigrant mothers^33^. Maternity care assistants and health practitioners implement adaptable strategies that embrace various cultural backgrounds and traditional customs, hence improving patient participation and support^34^. This change highlights the flexibility of multidisciplinary teams in addressing individual population demands, which is increasingly essential in varied healthcare settings.

Ultimately, continuous education and training for healthcare workers are fundamental for facilitating efficient multidisciplinary teamwork. Highlighting interdisciplinary knowledge facilitates enhanced care pathways in postnatal health^35^.

In conclusion, comprehensive and integrated postnatal care, based on a multidisciplinary approach, has the potential to markedly enhance health outcomes for mothers and infants, underscoring the importance of collaboration among diverse health professionals in providing thorough care during this crucial phase.

### Proposals for improving the postnatal care service

The primary proposals for improving postnatal care services from this study are as follows: 1) Implementation of home visits, as attending the local facility may stress the mother with a newborn; 2) Forums to facilitate dialogue among new mothers and couples to foster the establishment of supportive friendships and a feeling of community essential for women; 3) Assessment of the pelvic floor in all new moms; 4) Dissemination of local services and advocacy for the professional role of FaCM; and 5) Training programs designed for all professionals engaged in the childbirth process to provide clear information particularly concerning breastfeeding and infant nutrition.

### Limitations

This study is the first qualitative research to explore FaCM perspectives on the provision of postnatal care in the Lombardy region. However, the transferability of results may be limited in terms of a national context due to different healthcare organizational structures at regional levels. In the present study, the research team members directly interacted with the participants and minimized the potential impact of their professional backgrounds, experiences, and preconceived beliefs on these experiences.

## CONCLUSIONS

This study highlights the challenges that FaCM face in the new setting of community care. Assistance in the postnatal period has been defined as the ‘Cinderella service’ because it is scanty and inadequate^8^ when compared to the attention to the health and well-being of the dyad during pregnancy and birth. The role of FaCM must now emphasize the necessity of continuity of care, acknowledging that postnatal care is significantly linked to providing services that are more sensitive to women’s needs. The efficacy of FaCM is improved by the formation of stable and consistently present work teams. Despite the challenges faced, participants acknowledged their role in community services, perceiving it as fundamental to midwifery, which ultimately strengthens the future health of individuals, families, and the broader communities.

## Data Availability

The data supporting this research are available from the authors on reasonable request.
